# TNFα Amplifies DNaseI Expression in Renal Tubular Cells while IL-1β Promotes Nuclear DNaseI Translocation in an Endonuclease-Inactive Form

**DOI:** 10.1371/journal.pone.0129485

**Published:** 2015-06-11

**Authors:** Dhivya Thiyagarajan, Ole Petter Rekvig, Natalya Seredkina

**Affiliations:** 1 RNA and Molecular Pathology Research Group, Department of Medical Biology, Faculty of Health Sciences, UiT The Arctic University of Norway, Tromsø, Norway; 2 Department of Radiology, University Hospital of North Norway, Tromsø, Norway; INSERM, FRANCE

## Abstract

We have demonstrated that the renal endonuclease DNaseI is up-regulated in mesangial nephritis while down-regulated during progression of the disease. To determine the basis for these reciprocal DNaseI expression profiles we analyse processes accounting for an early increase in renal DNaseI expression. Main hypotheses were that *i*. the mesangial inflammation and secreted pro-inflammatory cytokines directly increase DNaseI protein expression in tubular cells, *ii*. the anti-apoptotic protein tumor necrosis factor receptor-associated protein 1 (Trap 1) is down-regulated by increased expression of DNaseI due to transcriptional interference, and *iii*. pro-inflammatory cytokines promote nuclear translocation of a variant of DNaseI. The latter hypothesis emerges from the fact that anti-DNaseI antibodies stained tubular cell nuclei in murine and human lupus nephritis. The present study was performed on human tubular epithelial cells stimulated with pro-inflammatory cytokines. Expression of the DNaseI and Trap 1 genes was determined by qPCR, confocal microscopy, gel zymography, western blot and by immune electron microscopy. Results from in vitro cell culture experiments were analysed for biological relevance in kidneys from (NZBxNZW)F1 mice and human patients with lupus nephritis. Central data indicate that stimulating the tubular cells with TNFα promoted increased DNaseI and reduced Trap 1 expression, while TNFα and IL-1β stimulation induced nuclear translocation of the DNaseI. TNFα-stimulation resulted in 3 distinct effects; increased DNaseI and IL-1β gene expression, and nuclear translocation of DNaseI. IL-1β-stimulation solely induced nuclear DNaseI translocation. Tubular cells stimulated with TNFα and simultaneously transfected with IL-1β siRNA resulted in increased DNaseI expression but no nuclear translocation. This demonstrates that IL-1β promotes nuclear translocation of a cytoplasmic variant of DNaseI since translocation clearly was not dependent on DNaseI gene activation. Nuclear translocated DNaseI is shown to be enzymatically inactive, which may point at a new, yet unknown function of renal DNaseI.

## Introduction

Lupus nephritis is a prototype immune complex disease where antibodies to dsDNA play a central role [1–3]. Deposition of chromatin fragment-anti-dsDNA antibody complexes has been shown to be one of the core factors that impose progressive renal inflammation [4–9]. The origin of chromatin exposed in these immune complexes has for a long time been discussed but consensus has not been reached. The same lack of international consensus relates to how anti-dsDNA antibodies really exert their pathogenic potential [10].

Recent results from our studies on the pathogenesis of murine and human lupus nephritis have demonstrated that DNaseI, representing > 80% of the total renal endonuclease activity [11,12] is profoundly down-regulated when mild or clinically silent mesangial nephritis progresses into severe membrano-proliferative lupus nephritis [13–15].

DNaseI executes the initial degradation of whole chromatin in context of apoptosis and necrosis [16,17]. This is important to perceive, as the initial chromatin degradation is a prerequisite for other secondary endonucleases to digest the chromatin fragments into small oligo-nucleosomes (see e.g. [16,17] for review).

With low renal DNaseI enzyme activity, chromatin is not appropriately fragmented and is instead transformed into secondary necrotic chromatin unmasked from apoptotic blebs (reviewed in [18,19], see also [20–22]). Once exposed, these chromatin fragments may bind glomerular basement membranes (GBM) and the mesangial matrix [5,23] at high affinity as demonstrated in vitro by surface plasmon resonance analyses [6]. Whether chromatin is targeted by anti-dsDNA antibodies in situ or before chromatin accumulates in membranes and matrices is not yet determined. However, complex formation of antibodies and chromatin fragments seems to be a central event in progressive lupus nephritis [10]. Thus, to understand the transcriptional and molecular basis for progressive lupus nephritis means to comprehend the mechanisms of renal DNaseI regulation in context of mesangial nephritis.

Despite the fact that DNaseI was isolated and characterized more than 60 years ago by McCarty et al. [24], the basic mechanisms responsible for regulation of the enzyme expression is not resolved. Due to previous observations that renal DNaseI has a tendency to be increasingly expressed during mesangial nephritis [25], we propose the simple hypothesis that pro-inflammatory cytokines might up-regulate the renal DNaseI enzyme. In addition, since the anti-apoptotic tumor necrosis factor receptor-associated protein 1 (Trap 1) [26–28] may be inversely co-regulated by a process denoted transcriptional interference [29], up-regulation of DNaseI may in fact down-regulate Trap 1 in mesangial nephritis, and vice versa as has been indicated in studies in (NZBxNZW)F1 mice [29].

Transcriptional interference is defined as a direct negative impact of transcription of one gene on transcription of a second anti-sense gene provided that the two genes overlap with each other. This process implies that if one of the genes is increasingly transcribed, the opposite gene is blocked for transcription.

Trap 1 is encoded in the opposite direction of DNaseI on the opposite DNA strand, and the transcripts overlap in their 3’ untranslated regions (3’UTR) (see http://genome.ucsc.edu/, see [30] for gene maps). In this type of gene organisation, it is likely that co-expression of the genes will be precluded by transcriptional interference [31,32]. If we take this information into consideration this allows us to assume that expression of renal DNaseI and renal Trap 1 are *i*. both blocked if both receive a transcriptional stimulus; *ii*. that one gene is blocked for transcription if the other is transcribed in response to specific stimuli.

The hypotheses that incited this study were therefore three-fold; *i*. the mesangial inflammation induces an early secretion of pro-inflammatory cytokines which directly increase DNaseI protein expression in tubular cells; *ii*. the anti-apoptotic protein Trap 1 is down-regulated as a consequence of increased transcription of the DNaseI gene through transcriptional interference of the two genes; *iii*. up-regulation of DNaseI gene expression simultaneously translocates a variant of DNaseI into the nucleus. The latter hypothesis is based on published results (see e.g. [33]) that DNaseI can be found in cell nuclei in different tissue. The mechanism and biological meaning of translocated DNaseI is not understood, may have impact in nuclei beyond its endonucleolytic activity in context of apoptosis (present study).

The data demonstrate that DNaseI is up-regulated in human renal proximal tubule epithelial cells (RPTEC) in response to TNFα and hypoxia, and at the same time, Trap 1 protein expression is reduced in a possible accordance with the proposed gene interference regulation of the two genes. However, an inhibition of IL-1β by siRNA in TNFα-induced up-regulation of DNaseI restores expression of Trap 1 indicating that DNaseI and Trap 1 are also regulated by other processes than by a pure transcriptional interference alone. We also show that stimulation of RPTEC with TNFα and IL-1β promotes translocation of DNaseI into the nuclei. However nuclear DNaseI translocation in response to these stimuli is fully an IL-1β-depended process and TNFα translocates DNaseI into the nuclei indirectly through the effect of TNFα-induced up-regulation of IL-1β. Notably, appearance of DNaseI in the nuclei of RPTEC was not accompanied by apoptotic chromatin fragmentation or terminal deoxynucleotidyl transferase dUTP nick-end labeling (TUNEL) positive nuclei. These data strongly indicate that DNaseI has (an-) other yet undetermined function(s) than just the endonucleolytic activity linked to cell death.

## Materials and Methods

### Ethic statements

The mouse study was approved by The National Animal Research Authority (NARA) (approval ID: 07/11167, ID-178). A coherent analysis on renal biopsies taken from patients with lupus nephritis was approved by The Scientific Ethical Committee, Copenhagen, Denmark ((KF) 01-2006-7214). Informed written consent was given by the patients.

### Murine and human tissue samples

Female (NZBxNZW)F1 (BW) and female age-matched BALB/c mice were purchased from Jackson Laboratory, Bar Harbor, Main, USA. Mice were euthanized by CO2 suffocation. Renal tissue was collected every second week from the age of four weeks until development of end-stage disease in BW mice, clinically defined when severe proteinuria developed (≥20 g/L), as described [25]. Included in this study are 5 mice from the pre-nephritic mice (Group 1, having no glomerular deposits of chromatin or IgG), 5 mice with mesangial nephritis (Group 2, having mesangial deposits of chromatin-IgG complexes), and 5 mice with end-stage disease (Group 3, with deposits of chromatin-IgG complexes in mesangium and in glomerular basement membranes (GBM)). Renal biopsies from patients, taken for diagnostic purposes, were classified according to the International Society of Nephrology/Renal Pathology Society (ISN/RPS) [34], and used for baseline observations on expression of DNaseI and Trap 1, and on nuclear DNaseI. Entry criteria were fulfilment of the ACR classification criteria for SLE and clinical indication for renal biopsy.

As renal control samples included in this study, we collected morphologically unaffected cortical tissue immediately after kidney extirpation in context of surgical treatment of renal cancer. Procedure for collection of renal tissue is described in details [29].

### Proteins and antibodies

The recombinant proteins used in this study are human TNFα (T6674), purchased from Sigma Aldrich (St. Louis, Missouri, USA), while human IL-1β (201-LB 1), IL-6 (206-IL), IL-10 (217-IL-005) and human IFNγ (285-IF-100) were from R&D systems (Minneapolis, USA). Trap1 recombinant protein (H00010131-P01) was obtained from Abnova (Taipei, Taiwan) and used as reference protein in western blot analysis. Human recombinant DNaseI was obtained from AH diagnostics, (Oslo, Norway) and was used as the control endonuclease enzyme in gel zymography and reference protein in western blot. Caspase 3 control cell extracts (untreated and cytochrome c treated Jurkat cells) were obtained from cell signaling technology (Boston, USA). The following antibodies were used in this study: rabbit anti-DNaseI (sc30058), goat anti-Trap 1 (sc-69289) and rabbit anti-histone H1 (sc10806) antibodies from Santa Cruz (Texas 75220 U.S.A); rabbit anti-DNaseI (ab113241) antibodies from Abcam (Cambridge, UK); rabbit anti-DNaseI (LS-B4846/31015), mouse anti-caspase 3 antibodies from LifeSpan Biosciences, cleaved caspase 3 antibody (9661) from Cell Signalling technology (Seattle, USA) and rabbit anti-actin antibody (A2066) was from Sigma-Aldrich (St. Louis, MO). Alexa 595-conjugated anti-rabbit IgG and Alexa 488-conjugated anti-goat IgG secondary antibodies were from Santa Cruz Biotechnology. Horseradish peroxidase- (HRP-) conjugated rabbit anti-goat IgG, and goat anti-rabbit IgG were purchased from Invitrogen (California, USA) for western blot studies.

### Cell culture experiments

RPTEC (Clontec, Lonza, Basel, Switzerland) were grown in Clontec REGM BulletKit (CC-3190) containing renal epithelial cell basal medium with the following growth supplements: hEGF, Hydrocortisone, Epinephrine, Insulin, Triiodothyronine, Transferrin, GA-1000, and fetal bovine serum at 37°C in 95% humidified air and 5% CO_2_. The cells were grown to 80% confluence and were stimulated with TNFα (2.5, 5, 10, 20ng/ml), IFNγ (12.5, 25, 50, 100ng/ml), IL-β1 (1.25, 2.5, 5, 10ng/ml), IL-6 (5, 10, 20ng/ml), and IL-10 (5, 10, 20ng/ml). The cells were harvested at 12, 24, 48 hrs and analysed for DNaseI and Trap 1 mRNA and protein expression levels and for intracellular location (whether cytoplasmic or nuclear) of expressed proteins by semi-quantitative western blots, and by confocal microscopy. For experimental validation of responses to stimuli in RPTEC, known responder molecules were analysed by qPCR (see Table 1 in Results).

**Table 1:** Gene expression analysis of published responses to cytokines stimulation of RPTEC^#^.

Cytokines used for stimulation of RPTEC	Responder parameters^§^ in RPTEC					
	MMP2	MMP9	IL-6	IL-10	TNFα	IL-1β
TNFα (20ng)	0.65 (±0.08) ns	152 (±31.3) ***	9.2 (±1.3) ***	1.6 (±0,2) ns	60.4 (±6.9) ***	5.0 (±1.0) **
L-1β (2.5ng)	2.8 (±1.2) ns	8.7 (±2.2) *	22.7 (±3.5) **	449.9 (±60,8) ***	8.2 (±3.3) ns	39.1 (±10.1) *
IFNγ (50ng)^¶^	0.7 (±0.02) ns	1.8 (±0.9) ns	2.3 (±0.6) ns	Not detected	0.3 (±0.1) ns	7.9 (±2.6) ns
IL-6 (20ng)	0.6 (±0.1) ns	2.1 (±0.1) ns	0.7 (±0.05) ns	18.9 (± 6,9)*	0.6 (±0.2) ns	0.7 (±0.1) ns
IL-10 (20ng)	0.8 (± 0.07) ns	0.3 (±0.07) ns	1.0 (±0.2) ns	Not Detected	0.5 (±0.2) ns	6.5 (±4.8) ns

^#^Data are given as fold change (± SD) compared with expression in non-stimulated RPTEC.

*Significant up-regulation of mRNA level after 48 hrs of stimulation when compare to non-stimulated RPTEC *<0.05;

**<0.005;

***<0.0005,

ns: no significant up-regulation.

^§^Quality control analysis in RPTEC stimulated with various cytokines. The expected response for each stimulation: TNFα up-regulates MMP9 [52,53], IL-6 [54,55] and IL-1β [36]. Stimulation of RPTEC with IL-1β up-regulates IL-10 [56], IL-6 stimulation up-regulates IL-10 [57] and IL-10 stimulation up-regulates IL-1β though this was not significant in these experiments.

^¶^ IFNγ up-regulated CXCL10 to 1000 fold in RPTEC [58].

One-way ANOVA with Dunett post hoc test was used to assess the differences in mRNA expression levels with all concentrations used for each cytokine to stimulate RPTEC when compared to non-stimulated cells. In the table the results are shown only for one concentration of each cytokine.

### Exposure of RPTEC to hypoxia

RPTEC were incubated under normal condition (control experiment) and in hypoxia condition (2% O_2_) using HeraCell 150i CO_2_ incubator (Thermo scientific, Massachusetts, USA). Cells were harvested at 3, 6, 8, 14, 24, 36, 48 and 72 hrs respectively and analysed for DNaseI and Trap 1 mRNA and protein expression.

### Short interference RNA (siRNA) transfection

In this study, 2x10^5^ cells/well in 6-well dishes and 2x10^4^ cells/well in confocal plates were seeded 24 hrs before transfection. After 24 hrs the media was removed and transfection was proceeded with fresh media containing 20ng/ml TNFα without and with 20 nM smart pool IL-1β siRNA (Dharmacon) using Lipofectamine 2000 (Invitrogen). The cells were harvested after 48 hrs after transfection and analysed for IL-1β mRNA and protein expression, and for nuclear DNaseI translocation. siRNA against P62 was used as a positive control for transfection in separate wells.

### RNA isolation and cDNA synthesis

Total RNA from the stimulation experiments was isolated using TRIzol (Invitrogen, CA, US) as described by the manufacturer. The quality and concentration of extracted RNA were determined spectrophotometrically by Nano Drop (Nano Drop technologies, Wilmington, USA). RNA samples were reverse-transcribed with random primers using High Capacity cDNA Reverse Transcription kit (Applied Bio Systems, Foster City, USA).

### Gene expression analyses

Quantitative real time PCR (qPCR) was performed using ABI Prism 7900HT Sequence Detection System (Applied Bio Systems). Pre-designed FAM-labeled gene expression assays (Applied Bio Systems) were used in this study. The accession numbers for these primer-probes are given in Table 2. TATA binding protein (TBP) was used as endogenous control. The relative expression levels were calculated using the ddCT method. Murine data are given as fold change compared with transcription in 8-weeks old control (BALB/c) mice.

**Table 2:** Primers and probes used in this study from Applied Bio Systems and their accession numbers.

Primer-Probes	Accession number
DNaseI	Hs00173736_m1
TRAP1	Hs00212476_m1
TBP	Hs00427621_m1
FAS	Hs00236330_m1
TNFα	Hs00174128_m1
INFγ	Hs00989291_m1
IL-1β	Hs01555410_m1
IL-6	Hs00985639_m1
IL10	Hs00961622_m1
MMP2	Hs00234422_m1
MMP9	Hs00234579_m1
Cxcl10	Hs99999049_m1
DNaseI	Mm01342389_g1
TBP	Mm00446973_m1
Beta-Actin	Mm01205647_g1
TNFα	Mm00443258_m1
IL-1β	Mm99999061_m1
IFNγ	Mm01168134_m1
IL-6	Mm00446190_m1
IL10	Mm99999062_m1

### Western blot

For western blot analysis, RPTEC were harvested in lysis buffer (40mMTris, 2mM CaCl_2_, 2mM MgCl_2_, 0.2% Triton X-100 pH 7.6) or 10 mg of murine kidney was homogenized and cleared by centrifugation in the same buffer as described [14]. Samples were stored at -80°C. Protein concentration was determined using BCA assay kit as recommended by the manufacturer (Pierce Biochemicals, IL, US). Approximately 10μg normalized protein per well was loaded onto a 4–12% Nu PAGE Bis-Tris gel (Invitrogen). SDS/PAGE and western blotting were performed according to standard procedures. Membranes were blocked with 5% (w/v) skimmed milk and probed with the relevant primary antibodies. After incubation with a HRP-conjugated goat anti-rabbit IgG or a rabbit anti-goat IgG antibody (Invitrogen), binding was assayed by chemiluminescence detection. Determination of molecular weight was done using MagicMark XP molecular weight markers (Invitrogen). Western blot of actin was included to ensure equal loading of the samples onto the gel.

### Nuclear and cytoplasmic extraction of proteins from RPTEC

Nuclear and cytoplasmic extracts from RPTEC were isolated using NE-PER Nuclear and Cytoplasmic Extraction kit according to suppliers’ instruction (Fisher scientific, USA). Briefly 1x106 cells from both resting and TNFα (20 ng/ml) stimulated RPTEC were harvested using a Trypsin/EDTA buffer (Clonetech). The washed cells were incubated with CER reagent I on ice for 10 min, with CER reagent II for 1 min and centrifuged at 15000 x g for 5 min. The supernatant contained the clean cytoplasmic extract. The pellet was re-suspended in NER reagent and incubated on ice for 40 min with vortexing every 10 min. Then the cells were centrifuged at 23000 x g for 10 min and the collected supernatant contained the nuclear extract. Both cytoplasmic and nuclear extracts were stored at -80°C until use. Control analyses demonstrated that histone H1 is absent in the cytoplasmic, but present in the nuclear fraction (see Results).

### DNA isolation and electrophoretic analysis

Total DNA was isolated from RPTEC without and with TNFα- (20ng/ml) stimulation for 48 hrs by using GenElute Mammalian Genomic DNA Miniprep kit from Sigma Aldrich (St. Louis, Missouri, USA). One μg of total DNA was size-fractionated by gel electrophoresis in 1.5% SeaKem LE Agarose gel from Lonza (Rockland, ME USA) in Tris-acetate-EDTA buffer and visualized under UV illuminator using staining with Nucleic Acid Gel Stains GelRed 730–2958 (VWR International, Oslo, Norway). The 100 bp DNA ladder from New England BioLabs Inc (MA, USA) was used to determine size distribution similar to an apoptotic 200-bp DNA ladder. DNA isolated by EZ1 DNA Tissue Kit (Qiagen, Bergen, Norway) from murine spleen incubated 6 hrs in 5 μM camptothecin purchased from Sigma-Aldrich (St. Louis, MO) was used as a positive control for apoptotic DNA fragmentation.

### DNaseI gel zymography

DNaseI enzymatic activity in murine kidney homogenates was analysed by protein separation in 10% SDS-polyacrylamide gels containing 40μg/ml heat–denatured salmon sperm DNA (Invitrogen Corp, Carlsbad, CA) as described [25]. After electrophoresis, proteins in the gel were washed and re-natured in DNaseI reactivation buffer (20 mM Tris/HCl, pH 7.6, 5 mM CaCl_2_, 5 mM MgCl_2_) for 24 hrs, and stained with Nucleic Acid Gel Stains GelRed 730–2958 (VWR International, Oslo, Norway). The gel was visualized under UV illuminator and the enzyme activity was related to the molecular weight of the recombinant endonuclease DNaseI in the gel. Collection of kidney samples for zymography was performed as described before [25].

### Confocal microscopy

RPTEC were seeded in 8 well μ-Slide plates (IB80826) from Ibidi (Munich, Germany). The cells were fixed in 4% paraformaldehyde in PBS for 10 min on ice. The cells were permeabilized using 0.1% Triton X-100 (VWR International) in PBS for 5 min, followed by blocking in 3% BSA for 30 min and incubation with anti-Trap 1 and anti-DNaseI antibodies for 60 min and subsequently with Alexa 488 anti-goat IgG and Alexa 595 anti-rabbit IgG antibodies for Trap1 and DNaseI, respectively, for 30 min, as described before [29]. The protein expression patterns were analysed using Zeiss-LSM510 Meta confocal microscopy. DAPI counter stain (Abbott Molecular Inc, USA) was used to stain nuclei.

### TUNEL Assay

The TUNEL kit was purchased from Roche (Indiana, USA) and the assay was performed according to the manufacturer’s instruction. Briefly, sham-stimulated and TNFα-stimulated RPTEC cells were fixed in 4% paraformaldehyde in PBS for 10 min on ice. The cells were permeabilized using 0.1% Triton X-100 (VWR International) in PBS for 5 min. The cells were subsequently treated with TUNEL Mix containing TUNEL label (11767291910; Roche) and the terminal deoxynucleotidyl transferase enzyme (11767305001; Roche), and incubated at 37°C for 1hr. The cells were washed with 1xPBS and nuclei were stained by DAPI and subsequently subjected to confocal microscopy using the Carl Zeiss LSM 700 confocal microscopy. As a positive control in the TUNEL assay, RPTEC were exposed to 10U recombinant DNaseI to induce single-stranded (TUNEL-positive) nicks, and the negative control was represented by TUNEL in absence of terminal deoxynucleotidyl transferase.

### IHC Analysis

Immunohistochemical staining of DNaseI was performed as described in detail [14] using EnVision+ System–HRP (DAB) (Dako Norge, Oslo).

### Immune electron microscopy

Immune electron microscopy (IEM) was performed to detect IgG deposits in glomerular matrices and membranes [25] and to detect tubular cell expression patterns of Trap 1 and DNaseI in renal ultrathin sections from (NZBxNZW)F1 mice exactly as described recently [29].

### Mass spectrometry

Nuclear and cytoplasmic proteins from RPTEC lysates were loaded onto 4–12% NU-PAGE SDS PAGE and the gel was stained with commassie blue. The gel was then destained and the respective bands were cut and the gel pieces were subjected to gel reduction, alkylation, and tryptic digestion using 6 ng/μl trypsin (V511A, Promega, Wisconsin, USA) [35]. OMIX C18 tips (Varian, Inc., Palo Alto, CA, USA) was used for sample clean up and concentration. Peptide mixtures containing 0.1% formic acid were loaded onto a Thermo Fisher Scientific EASY-nLC1000 system and EASY-Spray column (C18, 2μm, 100 Å, 50μm, 15 cm). Peptides were fractionated using a 2–100% acetonitrile gradient in 0.1% formic acid over 50 min at a flow rate of 250 nl/min. The separated peptides were analysed using a Thermo Scientific Q-Exactive mass spectrometer. Data were collected in a data dependent mode using a Top10 method. The raw data were processed using the Proteome Discoverer 1.4 software. The fragmentation spectra were searched against the Swissprot SwissProt_2011_12 database using an in-house Mascot server (Matrix Sciences, UK). Peptide mass tolerances used in the search were 10 ppm, and fragment mass tolerance was 0.02 Da. Peptide ions were filtered using a false discovery rate (FDR) set to 1% for peptide identifications.

### Statistics

Data are presented as mean of 3 parallels (± SD). An unpaired t-test was performed to test differences between IL-1β siRNA transfected and non-transfected cells and a one-way ANOVA with Dunett post hoc test was performed to compare each time point at different concentrations of cytokines to non-stimulated cells. For each parameter; p<0.05 was considered significant. All observations were included and Spearman was used for significance testing.

## Results

### Characterization of renal DNaseI and Trap 1 expression patterns in (NZBxNZW)F1 mice during progressive lupus nephritis

**Fig 1:**
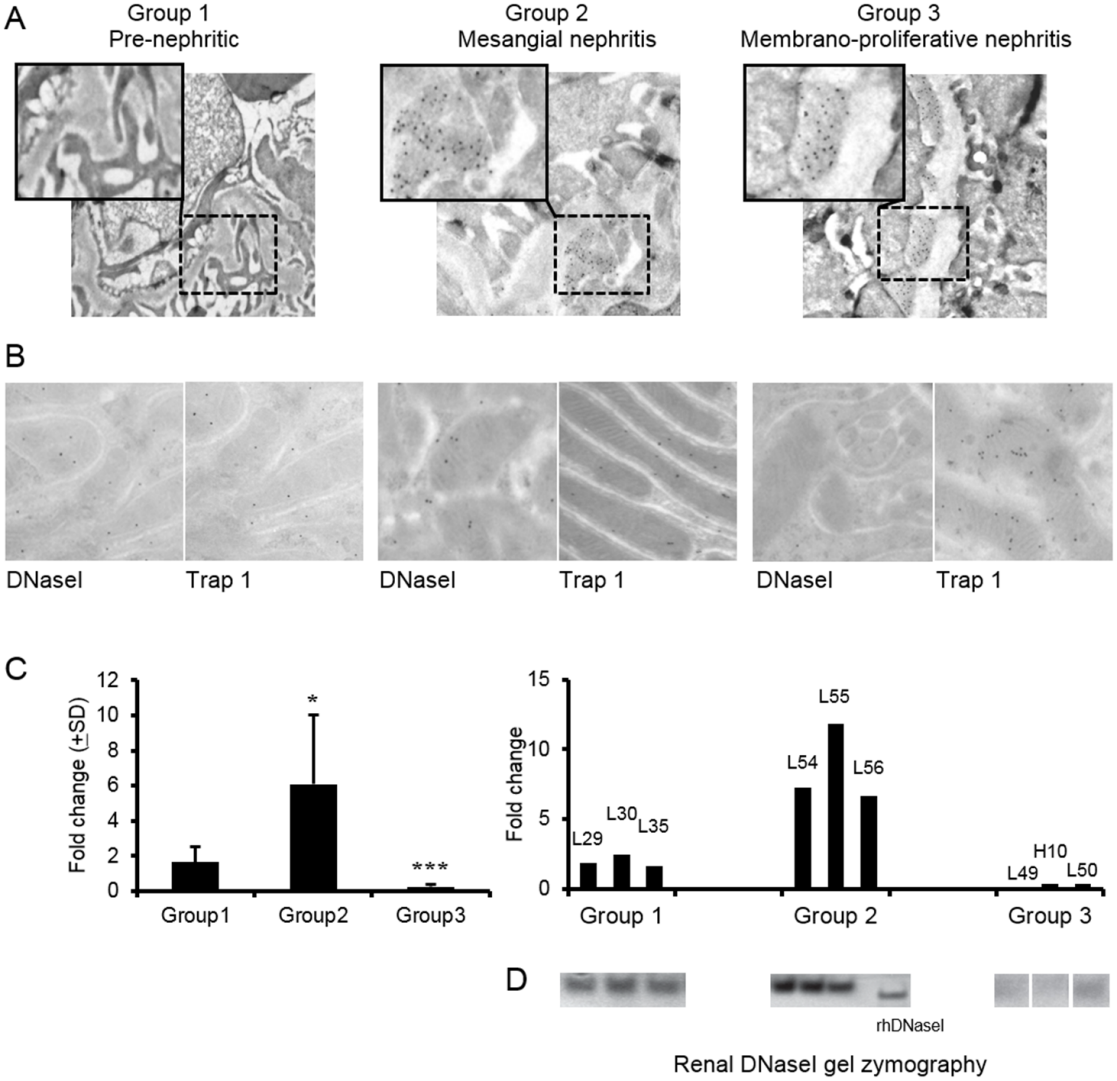
Renal expression of DNaseI and Trap 1 in (NZBxNZW)F1 mice during development of lupus nephritis. Mice were divided into 3 groups according to localization of electron dense structures (EDS) containing IgG (as traced by 5nm gold particles) in glomeruli as determined by immune electron microscopy (A). Pre-nephritic mice (Group 1) with no presence of EDS in glomeruli; mice with mesangial nephritis (Group 2) present EDS in the mesangium only; mice with membrano-proliferative nephritis (Group 3) have EDS both in the mesangium and in GBM (A). Expression patterns of DNaseI and Trap 1 were analysed by immune electron microscopy in Group 1–3 mice (B). DNaseI and Trap 1 were co-expressed in tubular cells in kidneys of Group 1 and Group 2 mice, while DNaseI staining was virtually absent in tubular cells in kidneys from Group 3 mice, while expression of Trap 1 was increased in these cells. The staining patterns were reflected by mRNA levels of renal DNaseI (C). In the left panel (C) average mRNA levels (±SD) are demonstrated in BW mice belonging to different groups (n = 5 per group). In the right panel (C), individual data on DNaseI mRNA levels in BW mice from Groups 1–3 (n = 3 per group) are demonstrated and compared with DNaseI gel zymography data (D). Renal DNaseI zymography data from all mice sacrificed every second week from 4–40 weeks are published in [25]. Significances: *<0.05; ***<0.0005.

Baseline data for the BW mice included in this study have been published recently [25]. These mice are grouped according to morphology of the kidneys as determined by immune electron microscopy. Fig 1A demonstrates that Group 1 mice have no deposits of immune complexes in glomeruli; Group 2 mice have chromatin-IgG complex deposits, seen as electron dense structures (EDS) [5,25] in the mesangium; Group 3 mice have EDS in the mesangium and within the GBM. Individual mice from each group were further analysed for renal expression of DNaseI and Trap 1 by IEM. Fig 1B demonstrates that in Group 1 and Group 2 kidneys, DNaseI and Trap 1 are moderately expressed, while in Group 3 kidneys DNaseI expression could not be detected. Expression of Trap 1 in Group 3 mice was conversely strong compared with expression in Group 1 and Group 2 mice. As demonstrated in Fig 1C (left panel), renal DNaseI mRNA varied considerably in mice analyzed at various stages of the disease progression (n = 5 per group). Notably, renal DNaseI mRNA levels were particularly high in Group 2 compared with the levels in Group 1 and Group 3 BW mice. In Group 3, DNaseI mRNA levels were hardly detectable (Fig 1C, [25]). In the right panel in Fig 1C, DNaseI mRNA levels are demonstrated in individual mice from Groups 1–3 (n = 3 per group). Of particular interest is the observation of high to very high DNaseI levels in some Group 2 mice. Coherent results were obtained when comparing DNaseI mRNA levels and DNaseI enzyme activity (Fig 1C and 1D, respectively). This may mean that in Group 2 kidneys with mesangial nephritis, the inflammatory milieu is a candidate factor to explain early up-regulation of DNaseI. This hypothesis is subsequently tested in RPTEC by controlled in vitro experiments. Renal DNaseI zymography data from all mice sacrificed every second week from 4–40 weeks as part of a longitudinal study is recently published [25].

### Characterization of DNaseI protein expression pattern in resting RPTEC

**Fig 2:**
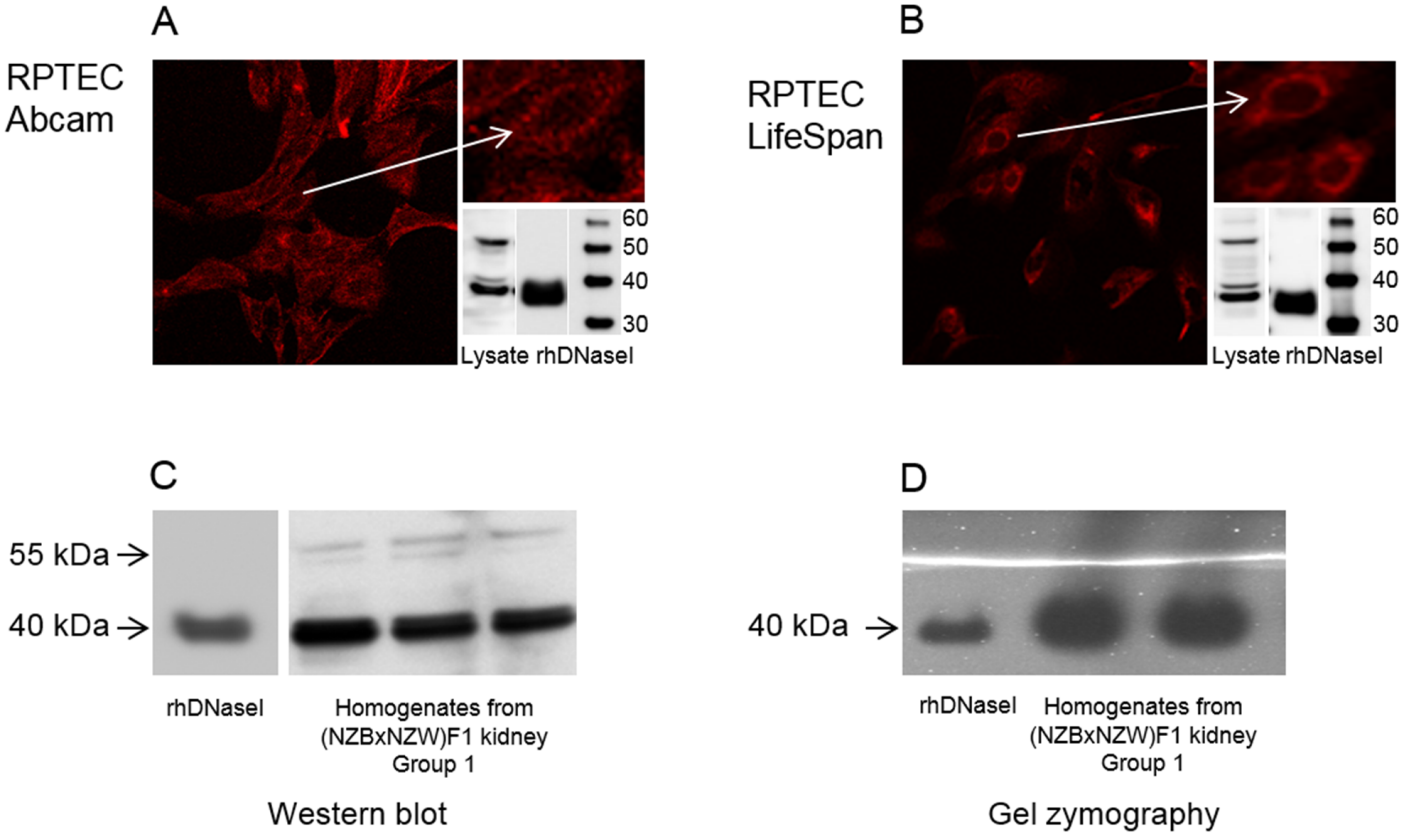
Characterization of DNaseI protein expression pattern in resting tubular cells and (NZBxNZW)F1 kidney homogenates. Patterns of immunofluorescence staining of human renal proximal tubule epithelial cells (RPTEC) and western blot analyses of RPTEC lysates are demonstrated (A and B). Anti-DNaseI antibodies from Abcam (A) and LifeSpan (B) predominantly stain cytoplasm and recognize two dominant bands: a strong 40 kDa band and a weaker 55 kDa band by western blot (A, B). The same bands were detected in kidney homogenates isolated from pre-nephritic BW mice Group 1 (C). As demonstrated by zymography only the 40 kDa bands possessed DNaseI enzyme activity while no enzymatic activity was found in area corresponding to the 55 kDa band. Human recombinant DNaseI was used as a control for both western blot analysis and DNaseI zymography.

Published data on sub-cellular location of DNaseI are discrepant. Some describe DNaseI in the cytoplasm [16], others indicate a nuclear position of DNaseI [33]. In order to firmly analyse how pro-inflammatory cytokines affect DNaseI expression in RPTEC, we first characterized DNaseI expression pattern by immunofluoresence and western blot in resting RPTEC. To perform the analysis, we tested three anti-DNaseI antibodies from different companies: Abcam, LifeSpan and Santa Cruz. All results with antibody from Abcam were reproduced with antibody from LifeSpan (Fig 2A and 2B respectively), while the antibody from Santa Cruz gave a different pattern in confocal microscopy as well as in western blot (S1A Fig) and were therefore excluded from this study.

Immunofluorescence analysis of DNaseI in resting RPTEC demonstrated staining in cytoplasm that was almost absent in the nuclei (Fig 2A and 2B). Western blot analysis of total cell lysate from RPTEC demonstrated two main bands recognized by the Abcam and the LifeSpan anti-DNaseI antibodies: a strong 40 kDa and a weak 55 kDa band (Fig 2A and 2B). The same results were observed in western blot analysis of kidney homogenates from BW mice (Fig 2C). To analyse if all bands were enzymatically active, DNaseI zymography was performed on murine kidney samples (Fig 2D). Only the 40 kDa band possessed DNaseI enzyme activity in the kidney samples. No enzymatic activity was found in the area corresponding to molecular weights above 40 kDa, and particularly not around 55 kDa (Fig 2D).

In order to confirm that the 40 kDa and 55 kDa bands indeed represented DNaseI protein variants, mass spectrometry analysis of all bands in RPTEC lysates detected by the anti-DNaseI antibodies was performed. The data demonstrated that anti-DNaseI antibodies specifically detect a DNaseI protein in all band regions defined by western blot (Table 3). As demonstrated in the Table 3, the gel region corresponding to cytoplasmic 40 kDa DNaseI contained 2 peptides recognized also in the nuclear fraction of 52 kDa (YDIALVQEVR) and in the cytoplasmic fraction of 55 kDa (LLDNLNQDAPDTYHYVVSEPLGR) respectively.

**Table 3:** Mass spectrometry analysis of renal tubular cell lysates size-fractionated by SDS gel electrophoresis.

Gel regions as MW spans that are subjected to mass spectrometry	Bands recognized by anti-DNaseI antibody on western blot		Presence of DNaseI protein verified by mass spectrometry		Common DNaseI peptides identified by mass spectrometry	
	Nuclear extract	Cytoplasmic extract	Nuclear extract	Cytoplasmic extract	Nuclear extract	Cytoplasmic extract
38–40 kDa	No bands	40 kDa	No	Yes	Not detected	LLDNLNQDAPDTYHYVVSEPLGR YDIALVQEVR
50–55 kDa	52 kDa	55 kDa	Yes	Yes	YDIALVQEVR	LLDNLNQDAPDTYHYVVSEPLGR

### The renal mRNA expression levels of pro-inflammatory cytokines in progressive murine lupus nephritis

**Fig 3:**
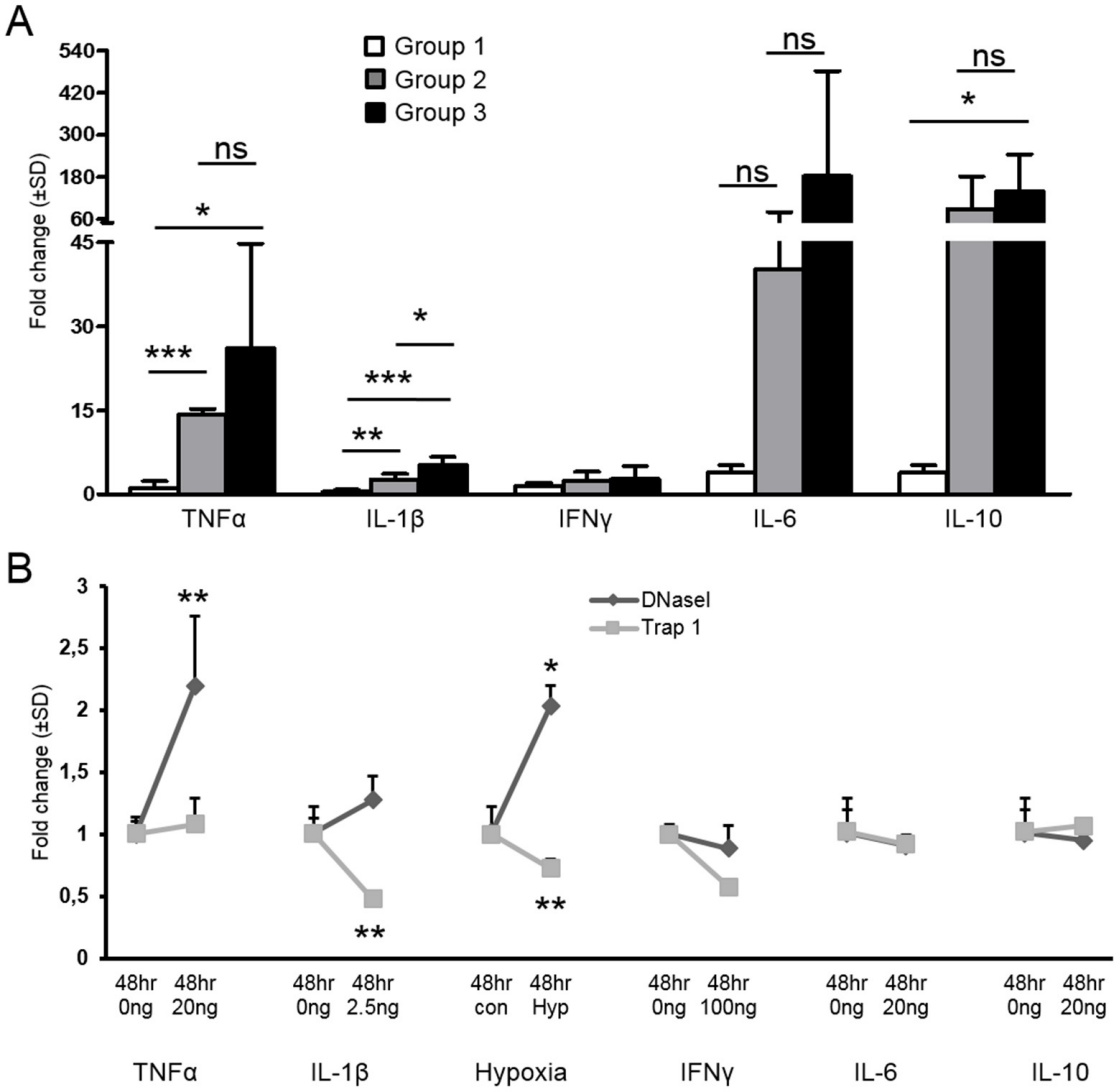
Pro-inflammatory cytokines profile in lupus nephritis and their effect on DNaseI expression in tubular cells. Renal mRNA expression of pro-inflammatory cytokines in (NZBxNZW)F1 mice during development of lupus nephritis demonstrated in A. The data demonstrate significant up-regulation of TNFα and IL-1β in Group 2 and Group 3 mice when compared to Group 1. IL-10 mRNA levels were significantly increased in Group 3 BW mice compared to Group 1. One-way ANOVA with Dunett post hoc test was performed to assess the difference in gene expression between the groups when compared to group 1 and 2. Effect of pro-inflammatory cytokines and hypoxia on DNaseI and Trap 1 mRNA levels in human renal proximal tubule epithelial cells (RPTEC) are shown in B. Significant elevation of DNaseI mRNA levels in RPTEC was found only upon TNFα stimulation and stress by hypoxia (B). No change in DNaseI gene expression was observed when RPTEC were stimulated by IL-1β, IFNγ, IL-6 or IL-10 (B). Trap 1 mRNA was significantly reduced after stimulations with IL-1β and hypoxia. Significances: *<0.05; **<0.005; ***<0.0005.

Analyses by qPCR were performed to quantify renal mRNA expression levels of pro-inflammatory cytokines in different stages of lupus nephritis in BW mice (Fig 3A). The mRNA levels of TNFα and IL-1β were significantly increased in kidneys from Group 2 and Group 3 mice when compare with Group 1. While mRNA levels of TNFα in Group 2 and 3 did not markedly differ, expression of IL-1β was significantly increased in Group 3 compared to Group 2. Expression levels of INFγ were stable, while levels of IL-6 and IL-10 varied considerably in kidneys from individual mice within each group. The differences of the latter between the groups were not statistically significant. However, a statistical significance was found for mRNA levels of IL-10 when comparing Group 3 with Group 1 (Fig 3A).

### The effect of pro-inflammatory cytokines on DNaseI mRNA expression in RPTEC

To analyse whether pro-inflammatory cytokines can affect expression levels of DNaseI in tubular cells, in vitro experiments applied to RPTEC were performed. The mRNA levels of DNaseI from RPTEC stimulated for 48 hrs with pro-inflammatory cytokines or stressed by hypoxia are demonstrated in Fig 3B. As is evident, TNFα and hypoxia significantly up-regulated DNaseI mRNA levels in RPTEC. Similar results were obtained after stimulation with TNFα for 24 hrs (S2A and S2B Fig, for protein and mRNA expression, respectively). In contrast, IL-1β, IFNγ, IL-6 and IL-10 had no influence on DNaseI gene expression in the cells (Fig 3B). Except for IL-1β and hypoxia, Trap1 mRNA levels were largely un-affected in the stimulated RPTEC, in contrast to reduced protein levels in response to TNFα (see below).

### Renal DNaseI protein expression and sub-cellular location in lupus nephritis and in RPTEC stimulated with pro-inflammatory cytokines

**Fig 4:**
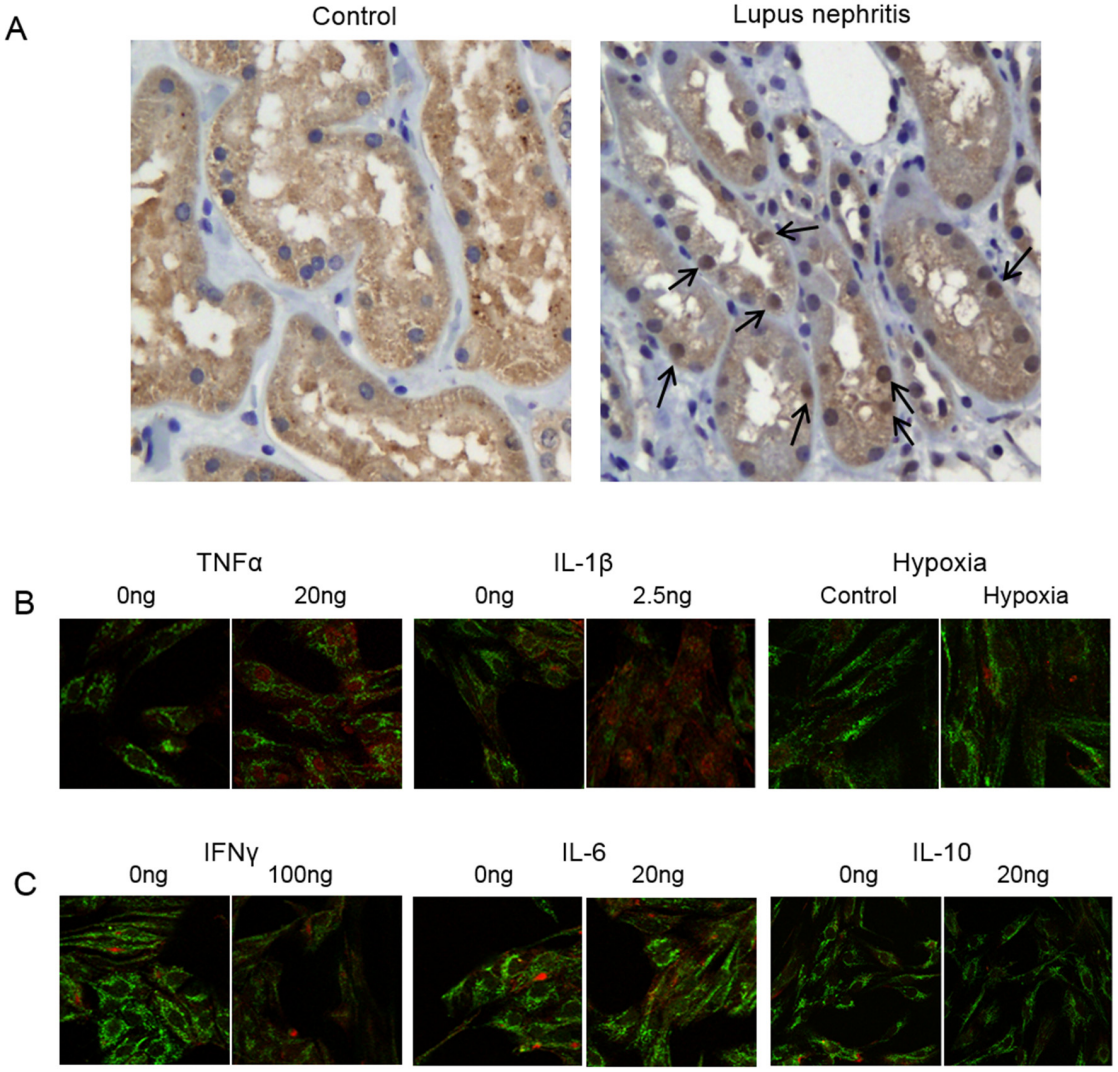
Sub-cellular location of DNaseI in lupus nephritis and in tubular cells stimulated with pro-inflammatory cytokines. Immunohistochemistry analysis of renal DNaseI in a control human kidney sample demonstrated that DNaseI was predominantly localized in cytoplasm and not in nuclei (A, left panel). In human lupus nephritis the DNaseI was abundantly present in both cytoplasm and notably in nuclei (A, right panel, arrows point at positively stained nuclei). In human renal proximal tubule epithelial cells (RPTEC) DNaseI protein expression pattern was determined together with Trap 1 by confocal microscopy (B, green: anti-Trap 1 antibody, red: anti-DNaseI antibody), only the merged pictures are shown. While resting cells expressed DNaseI predominantly in cytoplasm, stimulation of RPTEC by TNFα and IL-1β resulted in nuclear translocation of the protein (B). Interestingly, stress by hypoxia also resulted in increased expression of the DNaseI protein but not in nuclear translocation (B). Nuclear translocation of DNaseI was notably not observed after stimulation of RPTEC with IFNγ, IL-6 or IL-10 (C).

Analyses of renal DNaseI expression in murine and human lupus nephritis demonstrated that the cellular location of DNaseI differed during development of the disease. For example, in non-lupus kidneys DNaseI was expressed almost exclusively in cytoplasm, while in kidneys as having human lupus nephritis ISN/RPS class IV, DNaseI was abundantly present in tubular nuclei (Fig 4A). No morphological changes corresponding to apoptosis was observed in tubular cells with DNaseI nuclear staining (Fig 4A).

To experimentally analyse whether an inflammatory milieu may affect localization of the cellular DNaseI, RPTEC were stimulated with pro-inflammatory cytokines or stressed by hypoxia, and expression pattern and sub-cellular location of DNaseI was analysed by confocal microscopy (Fig 4B merged picture, green: anti-Trap 1 antibody, red: anti-DNaseI antibody). As demonstrated in Fig 4B, stimulation of RPTEC with TNFα or IL-1β for 48 hrs promoted DNaseI translocation into the nuclei. The same results were obtained at 24 hrs of stimulation (S2A, S2B, S2C and S2D Fig for TNFα and IL-1β respectively). The nuclear translocation of DNaseI reflected a specific process, since stressing the cells by hypoxia resulted in significant up-regulation of DNaseI mRNA levels (Fig 3B) and protein expression (S3 Fig), but not in nuclear translocation of DNaseI. Similarly, stimulation of RPTEC with IFNγ, IL-6 and IL-10 had no influence on DNaseI mRNA expression levels (Fig 3B) or sub-cellular protein location (Fig 4C). The experiments described here were validated by determination of known responses to the individual cytokines used as stimulators (Table 1).

### TNFα stimulation of RPTEC increases expression of DNaseI and translocates DNaseI into the nucleus

**Fig 5:**
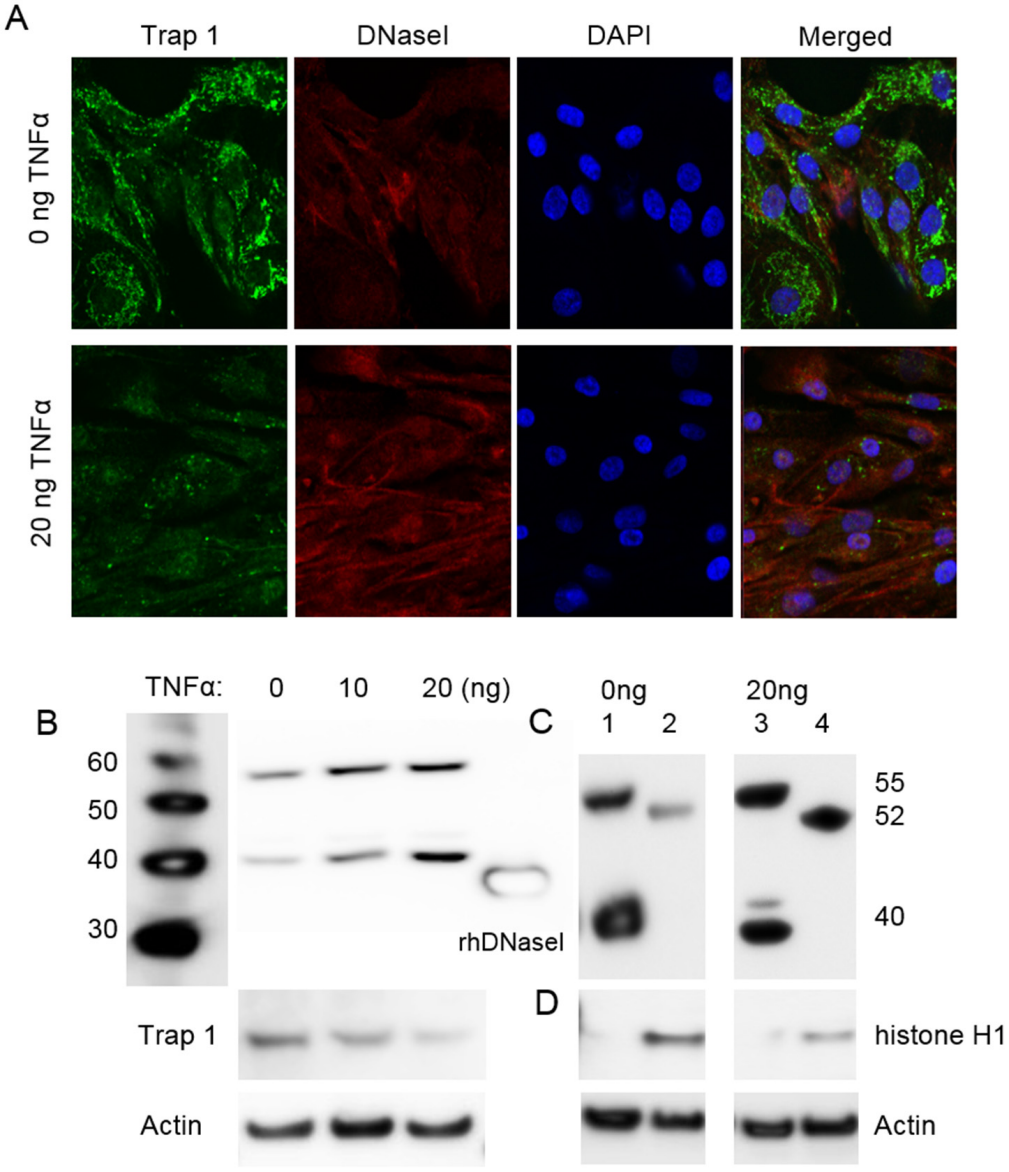
Cytoplasmic and nuclear DNaseI and Trap 1 expression in tubular cells after stimulation with TNFα. Confocal microscopy of sham-stimulated human renal proximal tubule epithelial cells (RPTEC) (A, upper panels), and cells stimulated with 20ng/ml of TNFα for 48 hrs (A, lower panels) was performed by using anti-Trap1 antibodies (A, green) and by anti-DNaseI antibodies (A, red). No nuclear staining of DNaseI was observed in sham-stimulation of the cells (A, upper panels). After stimulation of RPTEC with TNFα, expression of DNaseI generally increased in staining intensity, and DNaseI translocated into the nucleus (A, lower panels). Nuclear location of DNaseI was confirmed by co-staining with DAPI (A, blue). The data demonstrate that DNaseI and DAPI were in confocus (violet in the merged picture, A, lower panel), thus confirming that DNaseI indeed was translocated to the nucleus. Correspondingly, by western blots both the 40 kDa and the 55 kDa bands increased in response to increasing TNFα stimulation (0, 10, 20ng/ml TNFα) (B). In the same cells, Trap 1 expression was reduced after TNFα stimulation, as determined by confocal microscopy (A, lower panels versus upper panels, green staining) and by western blot (B). Nuclear and cytoplasmic protein fractions were isolated from RPTEC after 48 hrs stimulation by TNFα (C). The 40 kDa and 55 kDa bands were detected in cytoplasmic fraction (C, lane 1), while weak 52 kDa band was observed in nuclear fraction of sham-stimulated RPTEC (C, lane 2). After 48 hrs stimulation with 20ng of TNFα, an increased DNaseI expression in cytoplasm (C, lane 3) and remarkably strong expression of the 52 kDa DNaseI in the nuclei was apparent (C, lane 4). Thus data from western blot analysis corresponded with data from confocal microscopy with respect to TNFα-induced nuclear translocation of DNaseI. Only nuclear fractions of RPTEC contained histone H1 protein as shown in D, confirming controlled separation of nuclear and cytoplasmic fractions in the cells. Actin was used as a loading control in all western blot analyses.

RPTEC were stimulated for 24 hrs (S2A Fig) or for 48 hrs (Fig 5) by TNFα. In sham-stimulated RPTEC the anti-DNaseI antibody (Abcam) predominantly stained cytoplasm but not the nucleus (S2A Fig for 24h and Fig 5A for 48 hrs respectively, upper panels). In cells stimulated with 20ng/ml TNFα the anti-DNaseI antibody stained more strongly in cytoplasm and distinctly stronger in the nucleus (S2A Fig for 24 hrs and Fig 5A for 48 hrs respectively, lower panels). This nuclear translocation was consistent with an increased expression, not only of the 40 kDa protein, but also the approximately 55 kDa protein as demonstrated by western blot at 48 hrs of stimulation (Fig 5B). In agreement with data in Fig 1, Trap 1 expression was reduced concomitant with increased DNaseI expression (Fig 5B, see below). In order to characterize the sub-cellular origin of these proteins, we performed western blot analysis with anti-DNaseI antibodies on nuclear and cytoplasmic fractions of RPTEC (Fig 5C). The data demonstrated presence of a very weak 52 kDa band in the nuclear fraction of un-stimulated RPTEC (Fig 5C) but a very strong nuclear 52 kDa band after TNFα stimulation (Fig 5C). As is demonstrated, the 52 kDa protein was distinctly different from the 55 kDa protein in size.

To confirm nuclear location of DNaseI after stimulation with TNFα, nuclei of RPTEC were stained with DAPI (blue). As demonstrated in Fig 5A fluorescent signals from anti-DNaseI antibody (red) and DAPI (blue) were co-localized in the nuclei of RPTEC. Furthermore, western blot analysis of the histone H1 protein in the nuclear and cytoplasmic fractions of TNFα-stimulated RPTEC cell lysates demonstrated that the 52 kDa DNaseI band was exclusively present in the nuclear fraction that contained histone H1 (Fig 5D).

### Nuclear translocation of DNaseI in RPTEC depends on the effect of IL-1β

**Fig 6:**
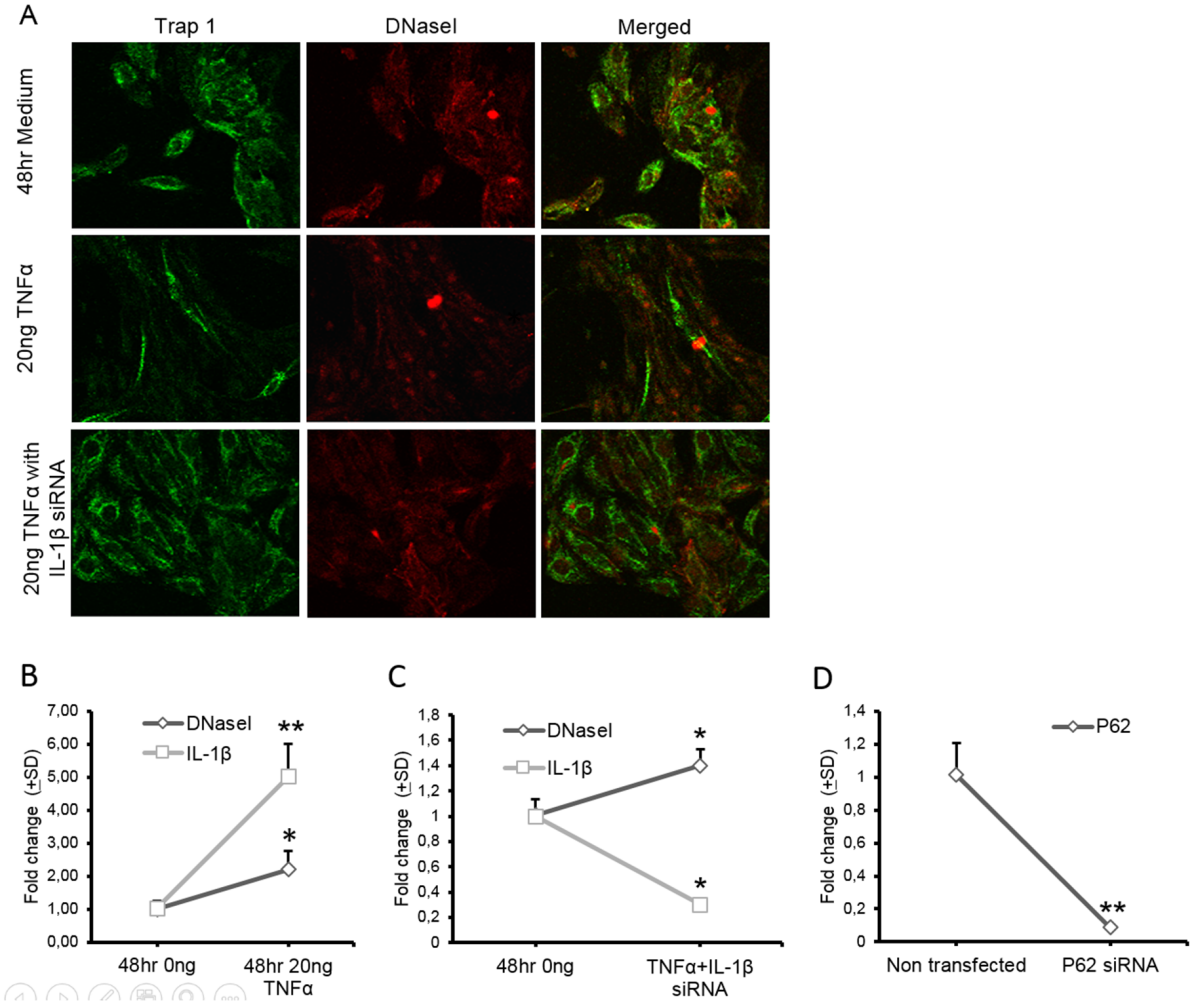
Effect of IL-1β siRNA on nuclear DNaseI translocation in RPTEC stimulated with TNFα. Confocal microscopy of sham-stimulated RPTEC (A, upper panels), cells stimulated with 20ng/ml of TNFα for 48 hrs (A, middle panels) and cells transfected with IL-1β specific siRNA and stimulated with TNFα for 48 hrs was performed by using anti-Trap1 antibodies (A, green) and by anti-DNaseI antibodies (A, red). While incubation of RPTEC with TNFα induced nuclear DNaseI translocation (A, middle panels), in RPTEC transfected with IL-1β siRNA and then stimulated with TNFα this did not result in translocation of DNaseI into the nuclei (A, lower panels). mRNA levels of DNaseI and IL-1β were increased in non-transfected RPTEC after stimulation with TNFα (B). Transfection with IL-1β siRNA inhibited mRNA level of IL-1β in RPTEC (C) but did not affect an increase in DNaseI gene expression induced by TNFα stimulation (C). Efficiency of the transfection procedure in RPTEC was controlled by transfection of P62 siRNA. As demonstrated in D, mRNA level of P62 was markedly decreased in transfected cells. Significances: *<0.05; **<0.005.

Translocation of DNaseI in the nuclei in RPTEC was demonstrated after stimulation with both TNFα and IL-1β. While TNFα up-regulates DNaseI expression and promotes nuclear DNaseI translocation (Figs 3B and 4B), IL-1β does not affect transcriptional or translational levels of DNaseI expression in RPTEC (Fig 3B) but still promotes nuclear DNaseI translocation (Fig 4B). Since TNFα is known to up-regulate expression of IL-1β [36], confirmed here to be valid also for tubular cells (Table 1), this opens for the possibility that TNFα translocates DNaseI into the nuclei indirectly through the effect of TNFα-induced up-regulation of IL-1β. We therefore transfected RPTEC with IL-1β siRNA and stimulated them at the same time with 20ng/ml of TNFα for 48 hrs (Fig 6A–6C). Although DNaseI expression was increased in TNF-stimulated and IL-1β siRNA transfected cells nuclear translocation of DNaseI did not occur in cells where expression of IL-1β was inhibited by siRNA (Fig 6A, lower panels). In TNFα stimulated RPTEC cells in absence of IL-1β siRNA transfection, DNaseI was notably translocated into the nuclei (Fig 6A, middle panels). In Fig 6D, a positive control for transfection is demonstrated in separate RPTEC cells with p62 siRNA. This treatment reduced the p62 mRNA significantly.

### IL-1β-dependent nuclear DNaseI translocation does not promote chromatin fragmentation, activation of caspase 3, or single-strand DNA cuts as determined by the TUNEL assay

**Fig 7:**
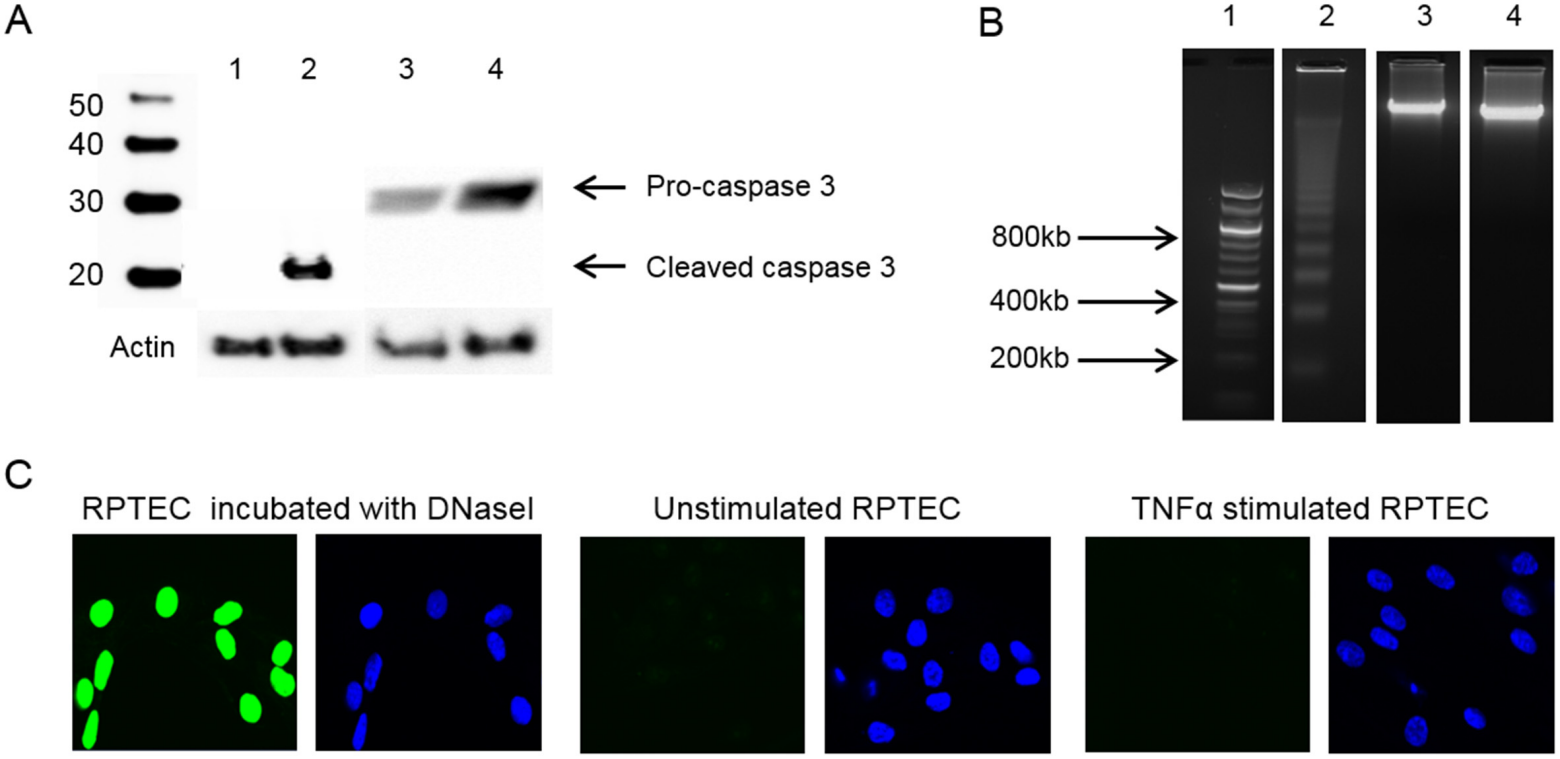
Nuclear DNaseI translocation in RPTEC was not associated with caspase 3 activation or DNA fragmentation. Western blot analysis was performed to detect presence of activated caspase 3 in cell lysates from RPTEC stimulated or non-stimulated with 20ng/ml of TNFα for 48 hrs (A). Resting Jurkat cells or cytochrome c treated Jurkat cells were used as a negative and positive control for activated caspase 3 (A, lane 1 and 2, respectively). As demonstrated in A, lane 4, TNFα-induced nuclear DNaseI translocation was not accompanied by activation of caspase 3 since only inactive pro-caspase 3 was found in RPTEC stimulated with TNFα. Only in cytochrome c treated cells activated caspase 3 was detected by western blot as shown in A, lane 2. Equal loading was controlled by staining with actin. Location of DNaseI in the nuclei of RPTEC did not result in chromatin fragmentation as demonstrated by DNA gel electrophoresis in B and TUNEL assay in C. DNA from murine spleen incubated with 5μM camptothecin for 6 hrs was used as a positive control for apoptotic DNA fragmentation (B, lane 2). No apoptotic DNA fragmentation was observed in DNA isolated from sham-stimulated RPTEC cells (B, lane 3) or in DNA isolated from RPTEC after stimulation with 20ng/ml of TNFα for 48 hrs (B, lane 4). TUNEL-positive staining in RPTEC cells was detected only after treatment with 10U of rhDNaseI (C, green). RPTEC cells stimulated with TNFα were TUNEL–negative as well as sham-stimulated cells as demonstrated in C. Lane 1 in B represent DNA size markers.

DNaseI can translocate into the nuclei upon apoptotic stimuli where it performs its canonical function- chromatin fragmentation [33,37]. However in RPTEC stimulated with TNFα or with IL-1β, nuclear translocation of DNaseI was not accompanied by induction of an apoptotic process or DNA degradation. As demonstrated in Fig 7A no activated caspase 3 was detected by western blot analysis in cell lysates of TNFα stimulated RPTEC. No DNA fragmentation was found in TNFα stimulated RPTEC with nuclear DNaseI location as shown by DNA agarose gel and by negative TUNEL assay (Fig 7B and 7C respectively). These data are in accordance with gel zymography analyses of murine nephritic kidneys, showing no endonucleolytic activity above 40 kDa (Fig 2D). These data indicate that DNaseI located in the nuclei after TNFα or IL-1β stimulations has another function than to serve as an endonucleolytic enzyme.

### Trap 1 expression in RPTEC stimulated with pro-inflammatory cytokines

In order to determine whether expression of Trap 1 would be affected by increased DNaseI expression due to a proposed gene interference mechanism, we analysed expression of Trap 1 and DNaseI in RPTEC stimulated with pro-inflammatory cytokines. The mRNA level of Trap 1 was found to be down-regulated in RPTEC in response to IL-1β stimulation for 48 hrs (Fig 3B) and hypoxia. Notably, the data demonstrate reduced Trap 1 protein expression in RPTEC upon TNFα stimulation for 48 hrs as shown by western blot analysis in Fig 5B and by confocal microscopy (Fig 5A, green). In accordance with this we have demonstrated that DNaseI and Trap 1 have an inverse protein expression pattern in BW mice (Fig 1B). Similarly, in RPTEC stimulated with TNFα, all of the variant DNaseI bands are increasingly expressed, and, at the same time, the Trap 1 protein expression is progressively reduced (Fig 5A and 5B). Interestingly, reduced expression level of Trap 1 protein during TNFα stimulation (Figs 5A and 6A, green) was restored when expression of IL-1β was simultaneously inhibited by IL-1β siRNA transfection (Fig 6A, green). This harmonizes with the fact that TNFα induces up-regulation of IL-1β in RPTEC (Table 1) and that IL-1β directly down-regulates Trap 1 mRNA (Fig 3B). It is also indicating that the regulation of Trap 1 and DNaseI during TNFα and IL-1β stimulations in the present cell experiments cannot be explained only by transcriptional interference related to the direct antagonistic expression effects of these proteins.

## Discussion

In this study we demonstrate 3 phenomena linked to expression of DNaseI. For the first, DNaseI is up-regulated most dominantly in early mesangial nephritis; secondly, experimentally induced over-expression of DNaseI in RPTEC translocates DNaseI into the nucleus, and the third, in human and murine lupus nephritis renal DNaseI is seen both in cytoplasm, as well as in the nuclei of tubular cells. Increased renal DNaseI expression in mesangial nephritis and nuclear DNaseI translocation had its experimental analogy when showing the same phenomena in tubular cells stimulated with TNFα and IL-1β, but not by hypoxic stress, nor by IFNγ, IL-6 or IL-10 in RPTEC. Therefore, up-regulation and nuclear translocation of the renal DNaseI protein is a highly selective response to specific stimuli.

At a certain time point following mesangial nephritis the renal DNaseI mRNA, DNaseI protein and its enzyme activity are inevitably lost. We have demonstrated that acquired silencing of the renal DNaseI gene expression appears to have a strong pathogenic impact on progressive lupus nephritis [12–14,25]. The clinical consequence of renal DNaseI gene silencing is a predictable transformation of mild mesangial lupus nephritis into end-stage organ disease (reviewed in [10]). However, an up-regulation of renal DNaseI precedes DNaseI gene silencing [25]. In this respect, three questions related to early lupus nephritis are regarded important to answer; *i*. Why is the renal DNaseI gene up-regulated during mesangial nephritis? *ii*. Is the term DNaseI unambiguously defined, or does DNaseI variants exist due to postsynthetic modifications that may allocate DNaseI in different cell compartments in context of its up-regulation? *iii*. Are all the variant of DNaseI proteins described here functional endonucleases?

Since there is a link between early mesangial nephritis and up-regulation of DNaseI, this may be caused by the local effect of pro-inflammatory cytokines. Consistent with this idea, stimulation of RPTEC with TNFα resulted in up-regulation of DNaseI, as shown by qPCR, western blot analyses, and by confocal immunofluorescence microscopy. At the same time, Trap 1 expression was reduced as demonstrated by western blots and confocal microscopy. Thus, increased expression of DNaseI seems to reduce expression of Trap 1 most probably by transcriptional interference of the two genes [29,31]. In an opposite context, these results may indirectly indicate that up-regulation of Trap 1 in kidneys may down-regulate DNaseI expression (experiments in progression).

There are few descriptive studies on gene regulation by transcriptional interference in clinical medicine. We have recently demonstrated an abrupt silencing of the DNaseI gene that correlated in time with up-regulation of Trap 1 in progressive lupus nephritis [29]. DNaseI is a pro-apoptotic protein, as it degrades chromatin in dying cells [16,17]. Trap 1 is an anti-apoptotic protein and is up-regulated during stress [27,38]. Trap 1 has anti-oxidant and anti-apoptotic functions [28,39]. It is up-regulated during stress and inflammation [26,27], and functions as a survival protein [40]. Trap 1 is up-regulated in different forms of cancers and overexpression corresponds to an unfavorable prognosis [26,41–44]. In support to a role of Trap 1 in cancer, studies based on oligonucleotide microarray demonstrated that Trap 1-positive cells contain high expression levels of genes that promote cell proliferation, compared with Trap 1-negative cells, which had correspondingly low gene expression levels [45]. Previously, we have shown that the ratio Trap 1/DNaseI was high to very high in Group 3 mice, where DNaseI was correspondingly very weakly expressed [29]. In a reciprocal situation, up-regulation of DNaseI may be followed by a down-regulation of Trap 1, in agreement with observations in this study.

Thus, the DNaseI and Trap 1 have opposite functions, as the first is involved in cell death, and the other in cell-survival, and they have reciprocal expression patterns [29]. Our preliminary data on reciprocal expression of the two genes [29] are in agreement with experimental data on transcriptional interference [31,46,47]. However, in this study we dissected the independent effects of TNFα and IL-1β on DNaseI and Trap 1 regulation. Our data indicate that the regulation of these two proteins in the TNFα induced system involves other signal molecules, like IL-1β. In this particular context DNaseI and Trap 1 regulation seems to be independent from the proposed transcriptional interference model.

If DNaseI has the potential to exert other functions than as a major endonuclease has not been determined. The results of studies on sub-cellular DNaseI location done by Peitsch et al. [37] and Rauch et al. [33] suggested that the enzyme is translocated into the endoplasmic reticulum, transported to Golgi apparatus and constantly secreted. Premature chromatin degradation due to accidental liberation of DNaseI from endoplasmic reticulum is protected by binding to cytoplasmic actin that inhibits DNaseI activity. Nuclear location of DNaseI was observed after induction of apoptosis. However, it is unclear how and why DNaseI reaches the nucleus. It was proposed to occur due to passive diffusion through the nuclear pores or through disrupted nuclear membranes during apoptosis [37].

In the present study we detected DNaseI in cytoplasm in resting RPTEC cell lines by confocal microscopy. Interestingly, western blot analysis from total cell lysates or from BW kidneys revealed two main protein bands: a strong 40 kDa and a weak 55 kDa band. Both of them were identified as DNaseI by mass spectrometry. However, only the band corresponding to 40 kDa was enzymatically active according to gel zymography on proteins extracted from BW kidneys. Additional studies are required to identify the structure and function of the 55 kDa DNaseI which is most probably a posttranslationally modified protein.

Stimulation of RPTEC with TNFα induced an increase of DNaseI protein expression and its nuclear translocation. Western blot analysis of total cell lysate demonstrated up-regulation of both 40 kDa and 55 kDa bands in RPTEC total cell lysate in response to TNFα. In order to identify the origin of the two different protein bands we isolated nuclei and cytoplasm from non-stimulated and stimulated RPTEC. Western blot analysis demonstrated that the 40 kDa and the 55 kDa protein bands were found in cytoplasm only. Notably, the nuclear variant, the 52 kDa DNaseI protein had a size distinctly different from the former two of 40 and 55 kDa. This nuclear-derived DNaseI was hardly detected in unstimulated cells but the expression of this latter protein was strongly increased after TNFα stimulation. Therefore, the 52 kDa DNaseI protein corresponds solely to nuclear translocation of DNaseI. Interestingly, the DNaseI translocation into the nuclei was not associated with increased DNaseI gene expression. This was demonstrated by two experiments. IL-1β itself translocated DNaseI, but did not up-regulate the DNaseI gene. This was confirmed when we simultaneously transfected the cells with IL-1β siRNA and stimulated them with TNFα. This manipulation resulted in up-regulation of the DNaseI gene expression, while the IL-1β siRNA prevented nuclear translocation. A firm conclusion is therefore that TNFα translocates DNaseI indirectly through the effect of TNFα-induced up-regulation of IL-1β [36]. These data allowed us to hypothesize that: *i*. De novo synthesized DNaseI induced by TNFα is not translocated into the nucleus; *ii*. The nuclear 52 kDa sized DNaseI is most probably an IL-1β-induced modification of the cytoplasmic 40 kDa DNaseI, and *iii*. If the 40 kDa DNaseI is transformed into the nuclear 52 kDa DNaseI, the modification transforms the active 40 kDa endonuclease into an endonuclease-inactive DNaseI protein with a yet unkown function (studies in progress).

Since TNFα is a known inducer of apoptosis, our observation on nuclear translocation of DNaseI one could interpret as a part of induced apoptotic cascade leading to apoptotic chromatin fragmentation. However, we did not observe signs of activated apoptosis in RPTEC upon TNFα stimulation as demonstrated by absence of apoptotic DNA fragmentation, TUNEL positive chromatin, activation of caspase 3 or morphological changes in RPTEC corresponding to apoptosis. As has been published before, use of TNFα alone is not sufficient to induce apoptosis in RPTEC in vitro unless the cells are simultaneously treated with RNA and protein syntheses inhibitors [48]. This is explained by the fact that TNFα triggers two distinct signalling pathways leading either to apoptosis or to activation of NF-κB transcription factors which will inhibit apoptosis through expression of anti-apoptotic genes [49–51]. On another hand translocation of DNaseI in the RPTEC nuclei which was not accompanied by apoptotic DNA fragmentation and appearance of TUNEL positive chromatin combined with the data from gel zymography demonstrating that only the 40 kDa DNaseI was enzymatically active allowed us to conclude that observed 52 kDa nuclear DNaseI in RPTEC had no enzymatic activity. The role of DNaseI variants and their translocation into the nucleus are real phenomena, while their function in the nucleus basically remains to be established. In this sense, this may open for new roles for DNaseI in cell biology. This is currently investigated in our laboratory.

## Supporting Information

S1 Fig DNaseI expression pattern in resting tubular cells by anti-DNaseI antibody from Santa Cruz. Patterns of immunofluorescence staining of human renal proximal tubule epithelial cells (RPTEC) and western blot analyses of RPTEC lysates are demonstrated. Anti-DNaseI antibodies from Santa Cruz predominantly stain nuclei in resting RPTEC and recognize two bands: 48 kDa and 50 kDa in western blot.
(TIF)

S2 Fig DNaseI and Trap 1 expression in tubular cells after 24 hrs stimulation with TNFα and IL-1β. Confocal microscopy of sham-stimulated human renal proximal tubule epithelial cells (RPTEC) (A,C, upper panels) and cells stimulated with TNFα for 24 hrs (A, lower panels) or with IL-1β (C, lower panels) was performed using anti-Trap1 antibodies (A and C, green) and anti-DNaseI antibodies (A and C, red). After TNFα stimulation, the expression of DNaseI was increased as demonstrated by anti-DNaseI antibodies on confocal microscopy (A, lower panel versus upper panel, red), while Trap 1 protein expression decreased during stimulation (A, lower panel compared with upper panel, green staining). Notably, we observed nuclear translocation of DNaseI staining in the TNFα stimulated cells also after 24 hrs (A, lower panel versus upper panel, C DNaseI is stained red)). The mRNA levels of DNaseI were increased while Trap 1 mRNA levels remained unaffected (B). Nuclear translocation of DNaseI was also observed after stimulation with IL-1β (C, lower panel versus upper panel, red), while mRNA levels of Trap 1 and DNaseI were not significantly changed at this time point (D).
(TIF)

S3 Fig DNaseI expression in RPTEC stressed by hypoxia and actin staining in RPTEC stimulated with TNFα. Western blot analysis of RPTEC lysates demonstrated a weak increase in DNaseI expression when comparing cells incubated at normoxia (lane 1) to cells after stress by hypoxia for 48 hrs (lane 2). After 72 hrs of hypoxia the expression level of DNaseI was markedly increased (lane 3 and lane 4 for normoxia and hypoxia respectively). Loading was controlled by actin staining.
(TIF)
