# Supplementary figures and images for: TNFα Amplifies DNaseI Expression in Renal Tubular Cells while IL-1β Promotes Nuclear DNaseI Translocation in an Endonuclease-Inactive Form
Source: PLoS One. 2015 Jun 11;10(6):e0129485. doi: 10.1371/journal.pone.0129485 (PMC4465975; doi:10.1371/journal.pone.0129485)

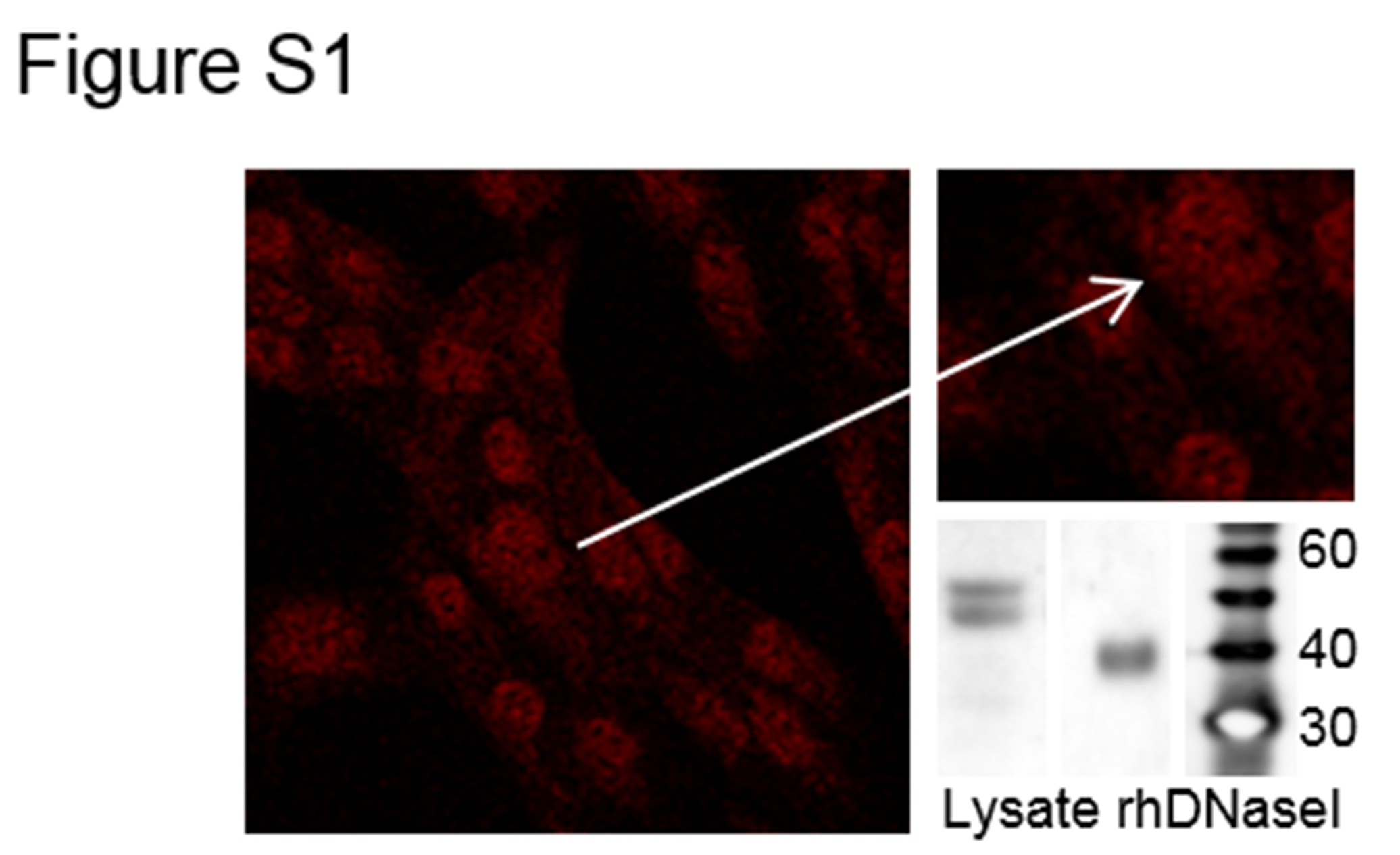

Supplement: S1 Fig — Patterns of immunofluorescence staining of human renal proximal tubule epithelial cells (RPTEC) and western blot analyses of RPTEC lysates are demonstrated. Anti-DNaseI antibodies from Santa Cruz predominantly stain nuclei in resting RPTEC and recognize two bands: 48 kDa and 50 kDa in western blot. (TIF) [file pone.0129485.s001.tif]

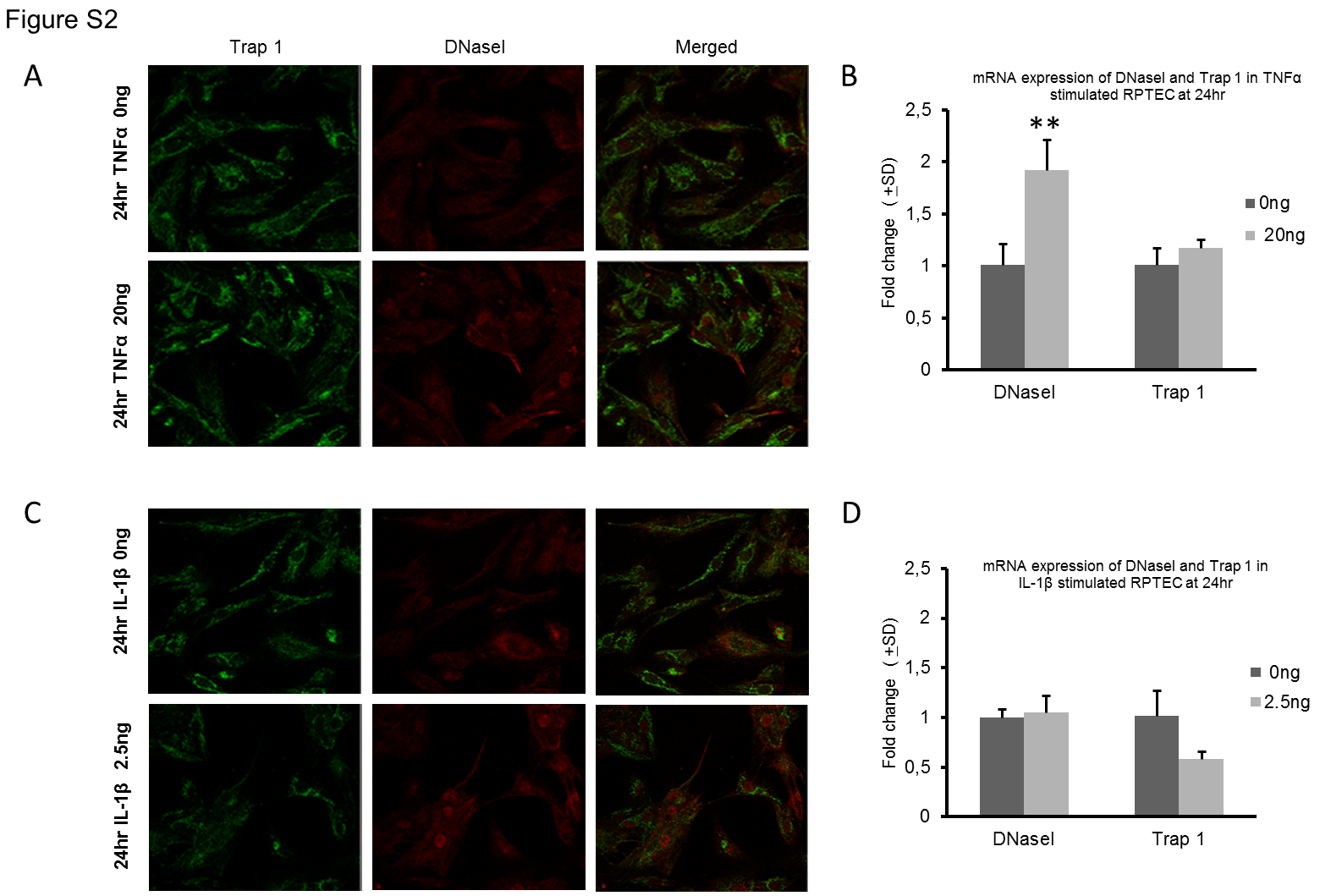

Supplement: S2 Fig — Confocal microscopy of sham-stimulated human renal proximal tubule epithelial cells (RPTEC) (A,C, upper panels) and cells stimulated with TNFα for 24 hrs (A, lower panels) or with IL-1β (C, lower panels) was performed using anti-Trap1 antibodies (A and C, green) and anti-DNaseI antibodies (A and C, red). After TNFα stimulation, the expression of DNaseI was increased as demonstrated by anti-DNaseI antibodies on confocal microscopy (A, lower panel versus upper panel, red), while Trap 1 protein expression decreased during stimulation (A, lower panel compared with upper panel, green staining). Notably, we observed nuclear translocation of DNaseI staining in the TNFα stimulated cells also after 24 hrs (A, lower panel versus upper panel, C DNaseI is stained red)). The mRNA levels of DNaseI were increased while Trap 1 mRNA levels remained unaffected (B). Nuclear translocation of DNaseI was also observed after stimulation with IL-1β (C, lower panel versus upper panel, red), while mRNA levels of Trap 1 and DNaseI were not significantly changed at this time point (D). (TIF) [file pone.0129485.s002.tif]

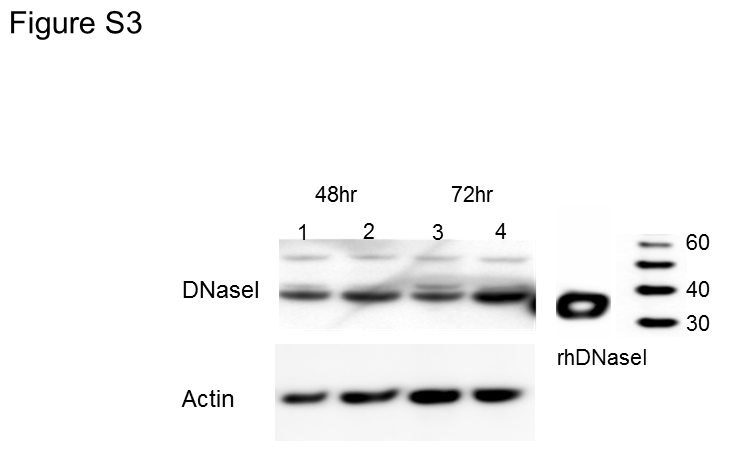

Supplement: S3 Fig — Western blot analysis of RPTEC lysates demonstrated a weak increase in DNaseI expression when comparing cells incubated at normoxia (lane 1) to cells after stress by hypoxia for 48 hrs (lane 2). After 72 hrs of hypoxia the expression level of DNaseI was markedly increased (lane 3 and lane 4 for normoxia and hypoxia respectively). Loading was controlled by actin staining. (TIF) [file pone.0129485.s003.tif]
